# Proposal for a revised Barthel index classification based on mortality risk assessment in functional dependence for basic activities of daily living

**DOI:** 10.3389/fpubh.2024.1478897

**Published:** 2025-01-14

**Authors:** Vicente Martín Moreno, María Inmaculada Martínez Sanz, Amanda Martín Fernández, Sara Guerra Maroto, Eva Sevillano Fuentes, Elena Pérez Rico, Irene Sánchez González, Miriam Fernández Gallardo, Julia Herranz Hernando, María Palma Benítez Calderón, Laura Calderón Jiménez, Elena Sánchez Rodríguez, Miguel Recuero Vázquez, Helena Alonso Samperiz, Irene León Saiz, Juana Marcos Guerra

**Affiliations:** ^1^Orcasitas Health Care Center, Madrid, Spain; ^2^i+12 Research Institute, Doce de Octubre Hospital, Madrid, Spain; ^3^Grupo Polibea Concierto, Madrid, Spain

**Keywords:** basic activities of daily living, instrumental activities of daily living, Barthel index, functional impairment, mobility, dependence

## Abstract

**Introduction:**

Functional dependence on the performance of basic activities of daily living (ADLs) is associated with increased mortality. In this study, the Barthel index and its activities discriminate long-term mortality risk, and whether changes in this index are necessary to adapt it to detect mortality risk is examined.

**Methods:**

Longitudinal study, carried out at the Orcasitas Health Center, Madrid (Spain), on the functional dependent population (Barthel ≤ 60). It included 127 people, with a mean age of 86 years (78.7% women and 21.3% men). Functional capacity was assessed using the Barthel index, and this index and each item it contains were analyzed as a test in relation to survival at three years, using tools that evaluate precision, discrimination, and calibration. The date of death was obtained from the health system.

**Results:**

Greater dependency to perform chair-to-bed transfers was associated with an increased mortality risk (HR 2.957; CI 1.678–5.211). Also, individuals with severe (HR 0.492; CI 0.290–0.865) and moderate (HR 0.574; CI 0.355–0.927) ADL dependence had a reduced mortality risk when more independent in chair-to-bed transfers. Among people with moderate ADL dependence, this percentage was 48%. Using dependence-independence for chair-to-bed transfer as a screening test for mortality, the test showed high sensitivity (0.91) and specificity (0.83), a positive likelihood ratio of 5.45, and a negative likelihood ratio of 0.11. The area under the ROC curve was 0.814 (CI 0.658–0.970; *p* = 0.001), with a *χ*^2^ = 0.235; *p* = 0.889, according to the Hosmer–Lemeshow test. The concordance C index was 0.814. According to Nagelkerke’s R^2^, the model explained 53.1% of the variance in survival. As a screening test, “chair-to-bed transfer” was superior to the Barthel index.

**Conclusion:**

ADL dependence for chair-to-bed transfers is an independent risk factor for mortality for any level of dependency. Therefore, a new classification of the Barthel index is proposed, in which “being dependent or requiring great assistance to perform chair-to-bed transfers” is considered severe dependence, even when the total score obtained via the Barthel Index is ≥40. We propose its use as a screening test in parallel to the Barthel index. The study suggests that the Barthel Index may have limitations in adequately discriminating mortality risk.

## Introduction

Compared with people of the same age without functional dependence, people with functional dependence for basic activities of daily living (ADLs) are associated with a lower life expectancy (1, 2). Its prevalence is higher in women (48.2%) than in men (26.6%), increasing as the population ages, and it also shows a socioeconomic gradient (3, 4). Functional ADL dependence is also one of the main causes of institutionalization (5).

The Barthel Index is the most widely used test for assessing functional dependence for ADLs (6). This index is an assessment tool composed of 10 essential daily living activities, such as feeding, mobility, and personal hygiene, measuring an individual’s functional dependence level. Over time, modifications of the Barthel Index have been proposed. In his proposal, Granger (7) increased the number of activities evaluated to 15, creating two differentiated indexes: a self-care index and a mobility index. Shah (8) kept the 10 assessed activities in his proposal but increased the number of response options for each activity.

Functional dependence has many facets, so the list of activities, or response options for each activity, could be substantially increased. However, in line with Granger’s proposal, two facets account for all of them.

On the one hand, the capacity for self-care may have more to do with quality of life than survival expectations. However, self-care is an indispensable facet of one’s role as a person, being able to take care of oneself versus being cared for, independence versus dependence.

On the other hand, mobility affects a person’s social capital and quality of life: the independence to continue to be who one truly is in the society in which one exists is important. When the loss of mobility affects a person’s ability to leave the house or causes them to be dependent on a wheelchair, it also affects their survival.

The Barthel Index has amply demonstrated its usefulness in the evaluation of patients beyond its initial indication, which was to assess functional capacity. The Barthel Index score is a good risk indicator of the probabilities of hospital admission (9), complications during surgery (10), complications at hospital discharge (11), mortality at hospital admission (12), and mortality at hospital discharge (13, 14). The Barthel Index score is also a good indicator of risk outside the hospital setting, both in predicting the risk of complications in numerous medical processes that do not require hospitalization (15, 16) or in the predictive evaluation of the development of long-term frailty (17), as well as in decision-making in relation to institutionalization (5, 18). It is a good predictor of mortality in all these situations (19, 20).

It is also a good indicator of risk outside the hospital setting, both in the evaluation of the appearance of complications in numerous medical processes that do not require hospitalization (15, 16) or in the predictive evaluation of the development of long-term frailty, as well as in decision-making in relation to institutionalization (5, 18).

On the other hand, other factors influence functional deterioration, such as residing in a socioeconomically disadvantaged environment (21). In these environments, moreover, small differences in personal economic level imply not only a higher level of functional dependence but also a lower probability of survival (22, 23). In addition, a higher level of functional dependence, a factor reflecting lower intrinsic capacity, has also been associated with higher mortality (23, 24).

Finally, mobility, evaluated as the difference between maintaining the ability to leave home or living homebound, also affects survival, and higher mortality has been observed among people living homebound (25). Living homebound is often the result of multiple factors, among which being a woman and having a severe functional dependency play a relevant role (26, 27).

Nevertheless, much remains to be evaluated. The aging of the population means that the number of people with functional dependence is likely to increase, a situation that will lead to a greater need and demand for care. Both factors will have a negative impact on the costs of care, both health and social (28). The social trend towards healthy aging makes it necessary to study which factors adopt patterns and design networks that lead to dependency (29). Pre-frailty and frailty associated with dependence to perform basic and/or instrumental activities of daily living are risk factors for mortality. Therefore, heuristics are needed to address these networks in the initial phases with the aim of preventing functional dependence (30). When dependence is established, try to slow its evolution (17, 31). The Barthel Index is a highly useful tool in this process.

However, the Barthel Index was not designed for these functions. Therefore, studies are needed to analyze the validity of the Barthel Index in these new areas and determine whether improvements in this index are needed, especially in the assessment of mortality risk. In this context, whether self-care activities should have the same weight as activities involving mobility in the assessment of mortality risk should be established. Moreover, there are patterns that create networks within functional dependence. These patterns could include various stages in functional deterioration, from being an independent person who can perform basic and instrumental activities to being bedridden and dependent, and where certain activities, such as chair-to-bed transfer, would represent a stage prior to being bedridden and differentiated from the rest of the activities of the Barthel index. These aspects are important not only in health planning but also in social services.

This study aims to evaluate the predictive capacity of individual components of the Barthel index as markers of mortality risk compared with the composite index, focusing on the “chair-bed transfer” component. In addition, we will analyze whether changes in the Barthel index are necessary in relation to this mortality risk. Finally, the association of these variables with the level of functional dependence, economic income, mobility, and mode of living in the community will be analyzed.

## Materials and methods

### Design and study population

This prospective longitudinal study was conducted between June 2020 and August 2023 at the Orcasitas Health Care Center, Madrid (Spain). Orcasitas is a peripheral neighborhood located in south Madrid. At the social planning level, Orcasitas is considered a socioeconomically disadvantaged neighborhood. This neighborhood comes mostly from the relocation of people who lived in substandard housing or shantytowns or from people who suffered immigration into Spain due to economic problems in the decades after the Civil War. These characteristics, together with a low cultural level, potentially increase the fragility of these people and predispose them to functional dependence, as described above.

Orcasitas has a single health center that serves its whole population, which, in June 2020, was 22,452 people. Care is provided in person, at the user’s request, at home, or by telephone. The health center has a services portfolio common to the entire National Health System. This portfolio of services includes protocolized activities, including a functional dependency screening for the entire population over 70 years of age. This screening is also carried out for individuals of other ages when there is clinical suspicion of functional deterioration and for all individuals between 65 and 70 years of age who have been assigned a high level of intervention owing to comorbidities.

**Figure 1:**
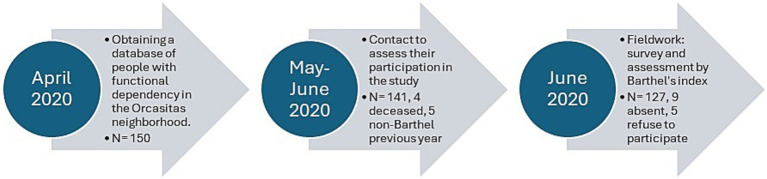
Flowchart of the process followed from the initial approach to carry out the study to the development of the fieldwork. Patient losses in the process and the cause of these losses are recorded.

The criteria for inclusion in the study were being 65 years of age or older and having a Barthel Index score ≤ 60 points, a score established as the cut-off point in the Protocol for the Care of the Dependent Person. The study included the entire non-institutionalized functionally dependent population of the Orcasitas neighborhood that was registered in the “Primary Care Scorecard: Subjective, Objective, Evaluation and Plan” (e-SOAP) application of the Health System in May 2020. There were 150 patients in the Orcasitas cohort. Exclusion criteria were not being at home during the study period (*n* = 9), a situation defined as being in another home as a consequence of the COVID-19 pandemic (*n* = 3), or being admitted to a hospital or nursing home (*n* = 6) during the study period, so was the absence of Barthel in the last year (*n* = 5). Five patients refused to participate in the study, and four died before the start of the study. Overall, 127 patients participated in this study. The process followed from the initial approach to carry out this study to the development of the fieldwork is represented in the flowchart shown in Figure 1.

### Data collection

Nurses who collected the data after the completion of the national confinement by COVID-19 in June 2020 received prior training to unify both the information conveyed to the participants and the possible answers to their questions. These nurses were unaware of the pre-confinement value of the patient’s Barthel index.

The data were obtained through a survey conducted by a trained nurse. The survey consisted of 48 questions. Participants were aware of the contents of the survey and could refuse to answer any question since confidential information was requested.

Functional dependence on the performance of ADLs was assessed via the Barthel Index. The data obtained were recorded in a database, and the score was classified according to the two most used methods. First, the score was classified according to the classic Barthel Index method, a method used by the Spanish National Health System’s Primary Care dependency protocol. This classification includes four levels of dependency: mild (Barthel Index score of 60 points), moderate (Barthel Index score of 40–55 points), severe (Barthel Index score of 20–35 points), and total dependency (Barthel Index score of <20 points).

Second, a second cut-off point was established at a Barthel Index score of 40 points, with two groups: severely dependent (Barthel Index score less than 40 points) and moderately dependent (Barthel Index score equal to or greater than 40 points). This criterion, which is also widely accepted among the scientific community, is recommended by the “Plan for attention to frailty and promotion of healthy longevity in the older adult in the community of Madrid” (32).

This procedure is justified by the coexistence of the two assessment systems in our practice, thus allowing better comparability and reproducibility of the results obtained in other studies.

The Individual Health Card (IHC) application was used to determine the income level of the population included in the study. This application establishes two categories: 1: an income level above 11,200 euros/year and 2: an income level below 11,200 euros/year. This cut-off point is established by the health system to differentiate the population according to economic income and to establish actions within health planning. An income level of less than 11,200 euros/year implies the receipt of free pharmacy services. This criterion is used as an indicator of the economic income level.

Orcasitas is a socioeconomically disadvantaged neighborhood, so the educational level was evaluated according to two categories: having received an education and not having received an education (illiterate; knowing how to read and write but not having received an education; and not having completed primary school).

The information systems of the Madrid Health Service were used to obtain the date of death of the participants who died during the three-year follow-up period. The professional who carried out this follow-up did not have access to the participants’ medical records or the data obtained from the surveys. At the same time, the health professionals participating in this study did not have access to the computer systems that recorded the date of death from the central services.

Finally, on the cohort, the effect of comorbidities, or of the social support associated with living independently or with others, has already been addressed in previous studies (22, 23). Therefore, this study will analyze this effect exclusively on the Barthel index activity or activities analyzed as a test to determine the risk of mortality. Following the criteria of the previously mentioned studies, five chronic diseases were taken as a cut-off point to evaluate the disease burden. Nutritional status was analyzed using the Body Mass Index (BMI). Finally, mobility was analyzed by means of instrumental activity, the ability to leave home, establishing a dichotomous assessment, leaving home versus living homebound.

### Data analysis

#### Descriptive analyses

The SPSS^®^ 18.0 statistical package was used to analyze the data obtained. The normality of the data distribution was checked via the Shapiro–Wilk test. Given the small sample size in the male group, less than 30, to reconfirm this equality of variances, and where necessary, ANOVA was performed with Levene’s homogeneity test, applying Welch’s correction as a robust test of equality of means. In this study, a value of *p* < 0.05 was considered significant.

The Barthel index consists of 10 activities. The following criteria were used to facilitate subsequent comparisons with other studies. To analyze the impact of each activity on survival and mobility, the traditional classification established by Barthel was used. In parallel, the influence of these activities was described in the text and tables, using both the traditional criteria and the subdivision of these activities according to the model proposed by Granger (7), which consisted of activities associated with mobility (chair-bed transfer, going up and down stairs, dressing-undressing, and walking) and activities associated with self-care (the rest).

Regarding missing cases, failure to answer four or more survey questions (10%) was considered equivalent to being excluded from the study. When the number of unanswered questions within the same questionnaire, or missing data within the same participant, was less than four, the method used was discarded by pairwise deletion.

### Bivariate comparisons

Differences between continuous variables were analyzed via Student’s t-test or the Mann–Whitney U test, and differences between categorical variables were analyzed using the chi-square test. The probability of the occurrence of an event was analyzed via odds ratios (ORs).

In addition, a bivariate analysis was performed with all the variables included in this study to identify those associated with long-term survival. Variables that showed significant association (*p* < 0.05) were included in the survival analysis via Cox regression and a proportional hazards model. The resulting model was summarized via the estimated coefficients, *p*-values, and hazard ratios (HRs) with 95% confidence intervals.

### Predictive models

The association between each of the activities included in the Barthel index and survival at three-year follow-up was analyzed using SPSS 18.0^®^. Following this analysis, the areas under the ROC curves for each of these activities were obtained. The selection criterion was an area under the curve (AUC) with statistical significance and a value greater than 0.700, with a lower confidence interval threshold greater than 0.500. Among those that satisfied this requirement, the activity that showed the highest AUC was selected, with the aim of determining the validity of its practical use as a test to detect the risk of mortality.

The selected activity was analyzed following two criteria: 1: using its extreme values, being independent versus being dependent for that activity. And 2: dichotomizing the activity, defining two groups: 1: “independent or requires minimal help” and 2: “dependent or requires great help.” A comparison was made as a diagnostic test against the Barthel index, and the same criteria were applied to this index in each case.

The Barthel index was used as the gold standard for comparing the risk of 3-year mortality associated with functional ADL dependence, a criterion justified by its recognized association with mortality, as reflected in numerous studies. The areas under the ROC curves of the two classifications of the Barthel index, classic four-level and summarized two-level, were obtained in relation to survival. At the same time, this procedure will make it possible to check whether the two methods of classifying functional capacity using the Barthel index are equivalent.

Other analyses were also performed. To analyze possible differences according to the level of dependence, the areas under the ROC curves of each activity included in the Barthel Index were also obtained for the groups of people with severe dependence and moderate dependence. The influence of the economic level on the results was also analyzed.

### Model validation

To measure functioning as a test to determine mortality risk, the activities of the Barthel Index that met the criteria described were subjected to a functional assessment. The measures used to assess functioning were as follows:

1. Measures that evaluate the accuracy of a prediction (the sensitivity, specificity, positive and negative predictive values, positive and negative likelihood ratios, and the posttest probability for both a positive and negative result) were determined via the statistical calculator of the Regional Health Service of Murcia (available at https://www.murciasalud.es/pagina.php?id=35022&idsec=2).
2. Measures that evaluate the discrimination capacity (the areas under the ROC curves and ORs) were obtained in relation to survival. The areas under the ROC curve were compared via the DeLong method with the Epidat 3.1 statistical package.
3. Measures that evaluate the calibration of the test (the model’s goodness of fit) were evaluated via binary logistic regression with the Hosmer–Lemeshow method. Using multinomial regression, Cox and Snell’s R2 and Nagelkerke’s R2 statistics were calculated to estimate the proportion of the variance in survival explained by the predictor variables. The concordance C index was also calculated to establish the degree of certainty that the test assigned a greater risk of a negative event (mortality) according to the ROC curve among participants with “dependence” in the Barthel Index activities who met the inclusion requirements. The C-index usually ranges between 0.6 and 0.85 in a prognostic risk model; the higher the value is, the closer it is to being a diagnostic criterion. Similarly, the Akaike information criterion (AIC) was used to identify the significant predictors in the model.

### Post-hoc analysis

Post-hoc analyses were performed to establish the percentages of variance in relation to survival explained by each variable, and predictive models were developed based on these results.

### Ethics statement

All participants were previously informed of the objectives of the study and the publication of the results. After agreeing to participate in the study, they signed the informed consent form, leaving a copy with the patient. The database was anonymized before data analysis.

This study was approved by the Local Research Commission of the Assistance Directorate Center, dependent on the Primary Care Management of the Department of Health of the Community of Madrid (Spain; resolution 16/20-C-Bis of June 29, 2020). The Ethics Committee of the Hospital Universitario Doce de Octubre endorsed this as sufficient approval (resolution 23/501 of September 26, 2023).

## Results

During the three-year follow-up period, 40.9% (*n* = 52) of the ADL-dependent individuals in this cohort died. Regarding the two levels of dependency for basic ADLs according to the abbreviated Barthel Index classification, 65.8% (*n* = 25) of the participants with severe dependence (*n* = 38) and 30.3% (*n* = 27) of those with moderate dependence (*n* = 89) died in 2020–2023 (HR 2,227; CI 1,514-3,276). However, among the participants who died, 48.1% (*n* = 25) had severe dependence for ADLs (Barthel Index score less than 40 points), and 51.9% (*n* = 27) had moderate dependence for ADLs (Barthel Index score of 40–60 points). According to the traditional classification of the Barthel Index, from 2020 to 2023, 78.9% (*n* = 15) of the participants with total dependence (*n* = 19), 52.6% (*n* = 10) of those with severe dependence (*n* = 19), 32.6% (*n* = 15) of those with moderate dependence (*n* = 46) and 27.9% (*n* = 12) of those with mild dependence (*n* = 43) died.

Functional dependence was assessed via the Barthel Index. This index consists of 10 items that assess various ADLs. When the association of each of these activities was analyzed with respect to survival at the three-year follow-up, “chair-to-bed transfer” was the activity that showed the highest association (Table 1). This activity also had the greatest area under the ROC curve (area 0.731; CI 0.642–0.820). Therefore, this Barthel Index activity was selected for specific analysis as a mortality risk factor.

**Table 1:** Activities of daily living assessed by the Barthel index and survival at the three-year follow-up in relation to the level of dependence for each activity.

The Barthel index activities and survival at the three-year follow-up					
Activity	Level	Alive	Deceased	Test	Statistic
Chair-to-bed transfers^1^	Unable	1 (1.3%)	10 (19.2%)	*χ*^2^ = 23.502	*p* < 0.001
	Major help	19 (25.3%)	23 (44.2%)		
	Minor help	30 (40%)	44 (34.6%)		
	Independent	25 (33.3%)	30 (23.6%)		
Dressing-undressing^1^	Dependent	14 (18.7%)	26 (50%)	*χ*^2^ = 15.170	*p* = 0.001
	Needs help	34 (45.3%)	18 (34.6%)		
	Independent	27 (36%)	8 (15.4%)		
Mobility on level surfaces^1^	Immobile or < 50 yards	10 (13.3%)	16 (30.8%)	*χ*^2^ = 10.554	*p* = 0.005
	Wheelchair independent or walks with help of one person	50 (66.7%)	34 (65.4%)		
	Independent	15 (20%)	2 (3.8%)		
Up and down stairs^1^	Unable	38 (50.7%)	41 (78.8%)	*χ*^2^ = 10.544	*p* = 0.005
	Needs help	24 (32%)	8 (15.4%)		
	Independent	13 (17.3%)	3 (5.8%)		
Feeding^2^	Unable	5 (6.7%)	12 (23.1%)	*χ*^2^ = 8.493	*p* = 0.014
	Needs help	11 (14.7%)	10 (19.2%)		
	Independent	59 (78.7%)	30 (57.7%)		
Bathing^2^	Dependent	47 (62.7%)	45 (86.5%)	*χ*^2^ = 8.766	*p* = 0.003
	Independent	28 (37.3%)	7 (13.5%)		
Grooming^2^	Needs help	29 (38.7%)	32 (61.5%)	*χ*^2^ = 6.436	*p* = 0.011
	Independent	46 (61.3%)	20 (38.5%)		
Bowels^2^	Incontinent	11 (14.7%)	15 (28.8%)	*χ*^2^ = 9.249	*p* = 0.010
	Occasional	12 (16%)	15 (28.8%)		
	Continent	52 (69.3%)	22 (42.3%)		
Bladder^2^	Incontinent	21 (28%)	23 (44.2%)	*χ*^2^ = 3.952	*p* = 0.139
	Occasional	39 (52%)	19 (36.5%)		
	Continent	15 (20%)	10 (19.2%)		
Toilet use^2^	Dependent	7 (9.3%)	23 (30.8%)	*χ*^2^ = 11.829	*p* = 0.003
	Needs some help	25 (33.3%)	44 (36.5%)		
	Independent	43 (57.3%)	17 (32.7%)		

1: tasks associated with mobility. 2: tasks associated with self-care. Both, according to Granger’s classification.

Participants fully dependent on ‘chair-bed transfers’ exhibited the highest mortality rate (90.9%), significantly higher than those requiring great assistance (54.8%), those who required minimal assistance (31.8%), or those who were independent (16.7%).

Within the group of people with total functional dependence (*n* = 19; Barthel Index score < 20 points), 36.8% (*n* = 7) were also dependent for “chair-to-bed transfers,” whereas the remaining 63.2% (*n* = 12) were not dependent for this activity. Among the participants in this group who died, 60% (*n* = 9) had total functional dependence but were not dependent on chair-to-bed transfers. The remaining 40% (*n* = 6) had total functional dependence and were dependent on other people to perform chair-to-bed transfers.

From 2020 to 2023, regarding the different activities included in the Barthel Index (Table 1), the mortality risk was not homogenous. The percentages of participants with dependence for performing each activity who died are as follows: feeding (70.6%), using the bathroom (69.6%), dressing-undressing (65%), mobility on level surfaces (61.5%), going up and down stairs (51.9%), bathing (48.9%), grooming (52.5%), bowel incontinence (57.7%), and bladder incontinence (52.3%); these percentages were lower than that observed among people with dependence for chair-to-bed transfers (90.9%).

Finally, among the participants who were dependent on others to perform chair-to-bed transfers, a high level of codependence was observed with respect to other activities of the Barthel Index. In relation to activities associated with self-care, the observed percentages of participants with dependency for each activity were 72.7% for “feeding,” 100% for “bathing,” 90.9% for “urinary incontinence,” 81.8% for “bathroom use” and “grooming,” and 72.7% for “fecal incontinence.” Among the activities associated with mobility, the percentages of participants were 100% for “mobility on level surfaces,” “up and down stairs,” and 90.9% for dressing-undressing (Table 1).

This implies a high mortality rate in persons who have a dependence on multiple activities. Regarding dependence on chair-to-bed transfers and dependence on another activity of the Barthel Index, the percentages of deaths associated with other activities were 87.5% (*n* = 7) for “feeding,” 90.9% (*n* = 10) for “bathing,” 88.9% (*n* = 8) for “bathroom use” and “grooming,” 90% (*n* = 9) for “urinary incontinence,” and 87.5% (*n* = 7) for fecal incontinence. Among the activities associated with mobility, the percentages of participants were 90.9% (10) for “mobility on level surfaces” and “up and down stairs” and 90% (*n* = 9) for dressing-undressing.

However, among participants who were not dependent on chair-to-bed transfers but were dependent on another activity, the risk of mortality was lower. Specifically, the percentages of deaths associated with self-care activities were 55.6% (*n* = 5) for feeding, 43.2% (*n* = 35) for bathing, 57.1% (*n* = 8) for bathroom use, 46.2% (*n* = 24) for grooming, 41.2% (*n* = 14) for urinary incontinence, and 44.4% (*n* = 8) for fecal incontinence. For activities associated with mobility, the percentage of deaths was 45.6% (*n* = 31) for going up and down stairs, 56.7% (*n* = 17) for dressing-undressing, and 40% (*n* = 6) for mobility on level surfaces.

In the second step, the use of the “chair-to-bed transfer” factor as a test for the assessment of mortality risk was analyzed. As a criterion for “healthy” or “not exposed,” the situation of “independently performing chair-to-bed transfers” was used. In addition, being “dependent for chair-to-bed transfers” was used as the criterion for “sick” or “exposed.” The outcome of the test was measured in terms of survival or death.

Analysis of the “chair-to-bed transfer” factor as a diagnostic test for mortality risk revealed that the test had a high sensitivity and specificity for predicting mortality risk and a high negative predictive value, with a positive likelihood ratio of 5.45 and a negative likelihood ratio of 0.11 (Table 2). These results translate to a predictive accuracy that can be considered “good.”

**Table 2:** Analysis of the “chair-to-bed transfer” indicator as a diagnostic test for mortality risk.

Evaluation of the Barthel Index and chair-to-bed transfer indicators^1^				
Chair-to-bed transfer and survival in 2023				
Chair-to-bed transfer	Status as of June 2023	Total dependence for chair-to-bed transfers	Independence for chair-to-bed transfer	Statistical test
	alive	1	25	*χ*^2^ = 13.964 *p* < 0.001
	deceased	10	5	

Evaluation of the chair-to-bed transfer indicator as a test of three-year mortality risk			
Test	Value	95% confidence interval	
		Lower limit	Upper limit
Sensibility	0.91	0.74	1.08
Specificity	0.83	0.70	0.97
Predictive value (+)	0.67	0.43	0.91
Predictive value (−)	0.96	0.89	1.04
Likelihood ratio (+)	5.45	2.40	12.41
Likelihood ratio (−)	0.11	0.02	0.72
Posttest probability (+)	0.85	0.71	0.93
Posttest probability (−)	0.10	0.02	0.42

The Barthel Index and survival in 2023				
Barthel index	Status as of June 2023	Total Dependency (Barthel Index score < 20 points)	Moderate Dependency (Barthel Index score of 60 points)	Statistical test
	Alive	4	31	*χ*^2^ = 13.964 *p* < 0.001
	Deceased	15	12	

Evaluation of the Barthel index as a three-year mortality risk test			
Test	Value	95% confidence interval	
		Lower limit	Upper limit
Sensibility	0.79	0.61	0.97
Specificity	0.72	0.59	0.85
Predictive value (+)	0.56	0.37	0.74
Predictive value (−)	0.89	0.78	0.99
Likelihood ratio (+)	2.83	1.66	4.82
Likelihood ratio (−)	0.29	0.12	0.72
Posttest probability (+)	0.74	0.62	0.83
Posttest probability (−)	0.23	0.11	0.42

Comparative analysis with respect to the Barthel Index (gold standard). 1: Analysis was performed using the following criteria: “Exposed”: independence for “chair-to-bed transfers” or a Barthel Index score of 60 points. “Not exposed”: dependence for “chair-to-bed transfers” or total dependence (Barthel Index score < 20 points). Positive test: death. Negative test: survival.

**Figure 2:**
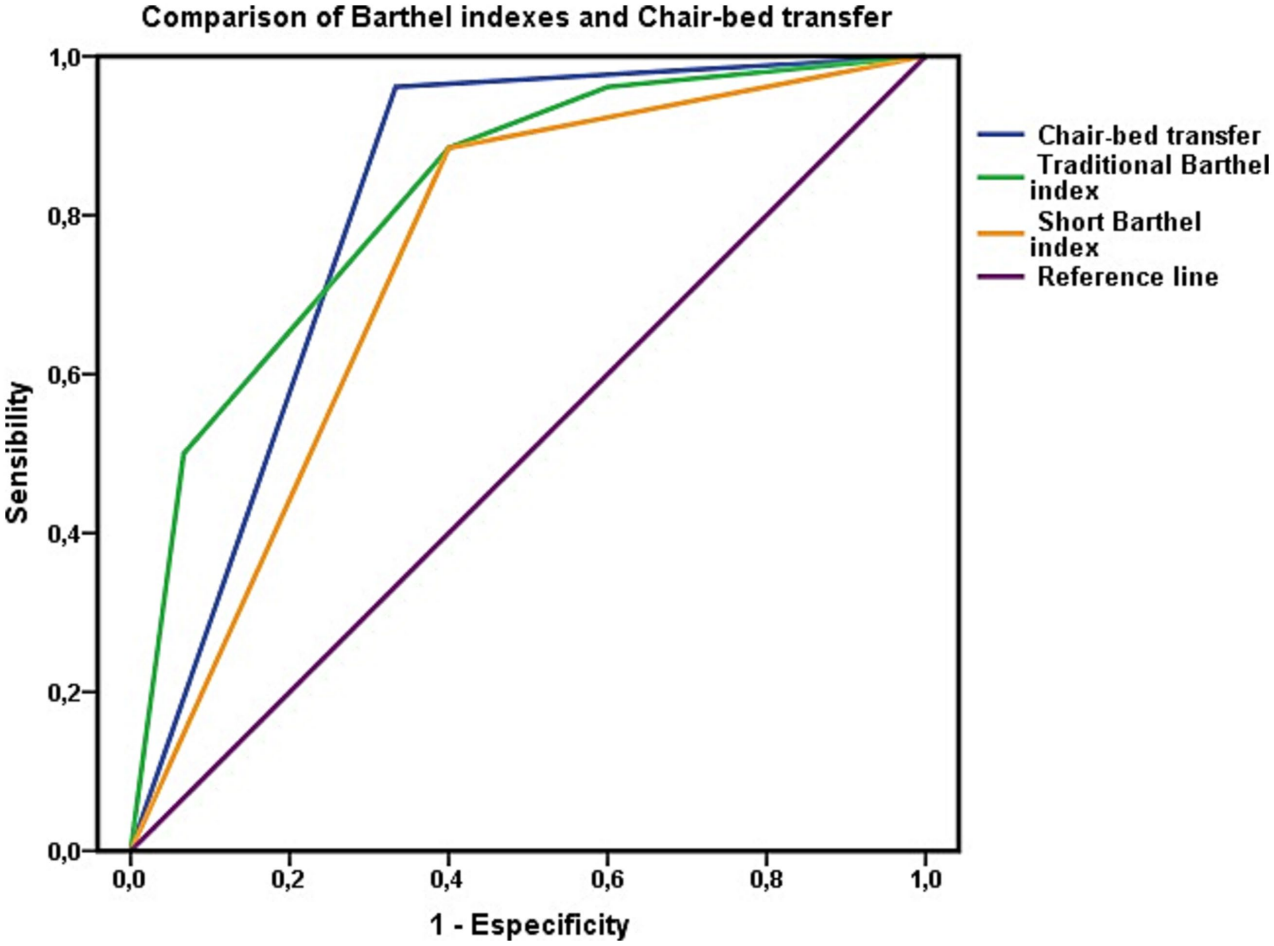
Evaluation of the ability to discriminate mortality risk using the area under the ROC curve for the “chair-to-bed transfer” factor. The areas under the ROC curve of the Barthel index in its traditional and summary versions represent the dependency to perform basic activities of daily living of people who are dependent or independent to perform the “chair-to-bed transfer.”

After performing this analysis, the discriminatory capacity of this factor as a diagnostic test was analyzed via the area under the ROC curve, which revealed an area of 0.814 (CI 0.658–0.970; *p* = 0.001; Figure 2). Compared with being dependent, being independent on others to perform “chair-to-bed transfers” was associated with a lower risk of mortality at the three-year follow-up (OR 0.020; CI 0.002–0.193). The result obtained indicated that their discrimination capacity was good.

Finally, the calibration of the test was evaluated via the Hosmer–Lemeshow test, which yielded a *χ*^2^ value of 0.235 (*p* = 0.889, very close to 1). The Cox and Snell R2 values showed that the model explained 38.8% of the variance in survival, whereas Nagelkerke’s R2 value explained 53.1% of this variance. The concordance C index was 0.814.

The capacity of the variable “chair-to-bed transfer” to assess mortality risk was contrasted with the gold standard, the Barthel Index score (Table 2). Following the same criteria as in the previous step, two levels of the Barthel Index were used for comparison. These levels were “total dependence” (Barthel Index score < 20 points), defined as “total dependence to perform chair-to-bed transfers,” and “mild dependence” (Barthel Index score of 60 points), defined as “independence to perform chair-to-bed transfers.” At the mild dependency level of the Barthel Index (Barthel Index score = 60 points), no person was dependent on “chair-to-bed transfers.”

**Figure 3:**
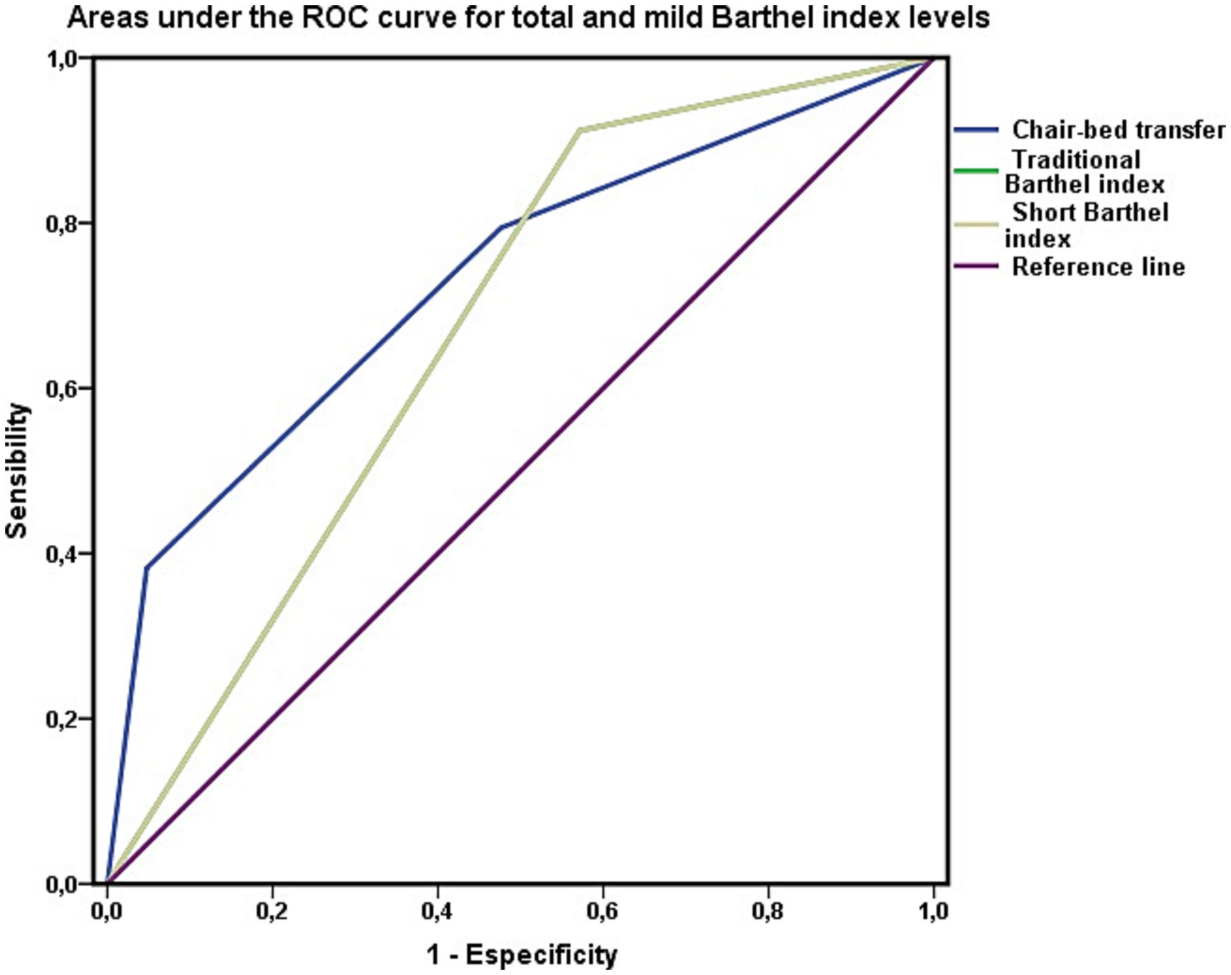
Barthel index as a test to discriminate mortality. Areas under the ROC curve of the total (Barthel <20) and mild (Barthel 60) levels of the Barthel index in persons not dependent for chair-to-bed transfer. The area under the ROC curve of the variable “chair-bed dependence” represents exclusively persons who require great help to perform this activity, require minimal help, or are independent in its performance.

In the determination of mortality risk, the Barthel Index has lower sensitivity and specificity and a high negative predictive value. The test revealed that the Barthel Index had a positive likelihood ratio of 2.83 and a negative likelihood ratio of 0.29, and its rating was regular. The accuracy of the index in predicting mortality risk was lower than that of the chair-to-bed transfer factor. In relation to the predictive ability of the index (Figure 3), the area under the ROC curve was 0.721 (CI 0.587–0.855; *p* = 0.001), so its discriminatory capacity was regular.

Finally, for the calibration of the Barthel Index, the Hosmer–Lemeshow test yielded the following results: *χ*^2^ = 0.149 and *p* = 0.928. The Cox and Snell R2 values showed that the model explained 27.2% of the variance in survival, whereas Nagelkerke’s R2 value explained 36.5% of that variance. The concordance C index was 0.792. These results revealed a lower performance of the Barthel index as a test for assessing mortality risk.

These results suggested that the Barthel Index performed worse in assessing mortality risk than the chair-to-bed transfer factor alone. However, when the areas under the ROC curve were compared via the DeLong method, the large difference observed between the areas calculated for both variables, the chair-to-bed transfer factor and the Barthel index, was not significant (*χ*^2^ = 0.171; *p* = 0.279). Thus, in terms of predictive capacity, we can only consider a trend, with the remaining parameters analyzed, marking the differences that may have clinical utility.

Along these lines, we visually analyzed the impact of the chair-to-bed transfer factor on the Barthel Index (Figures 2, 3). In Figure 2, the area under the ROC curve for the Barthel Index was calculated among people who were dependent on chair-to-bed transfer or independent of this activity. This figure thus represents the influence that dependence-independence for chair-to-bed transfers has on the Barthel index.

In Figure 3, the area under the curve for the Barthel Index was calculated among people who had total (Barthel Index score < 20 points) or mild (Barthel Index score of 60 points) functional dependence, without incorporating persons who were dependent for chair-to-bed transfers in this calculation. This represents the difference between being mildly or dependent according to the Barthel Index, discounting the effect of being dependent for chair-to-bed transfers.

**Figure 4:**
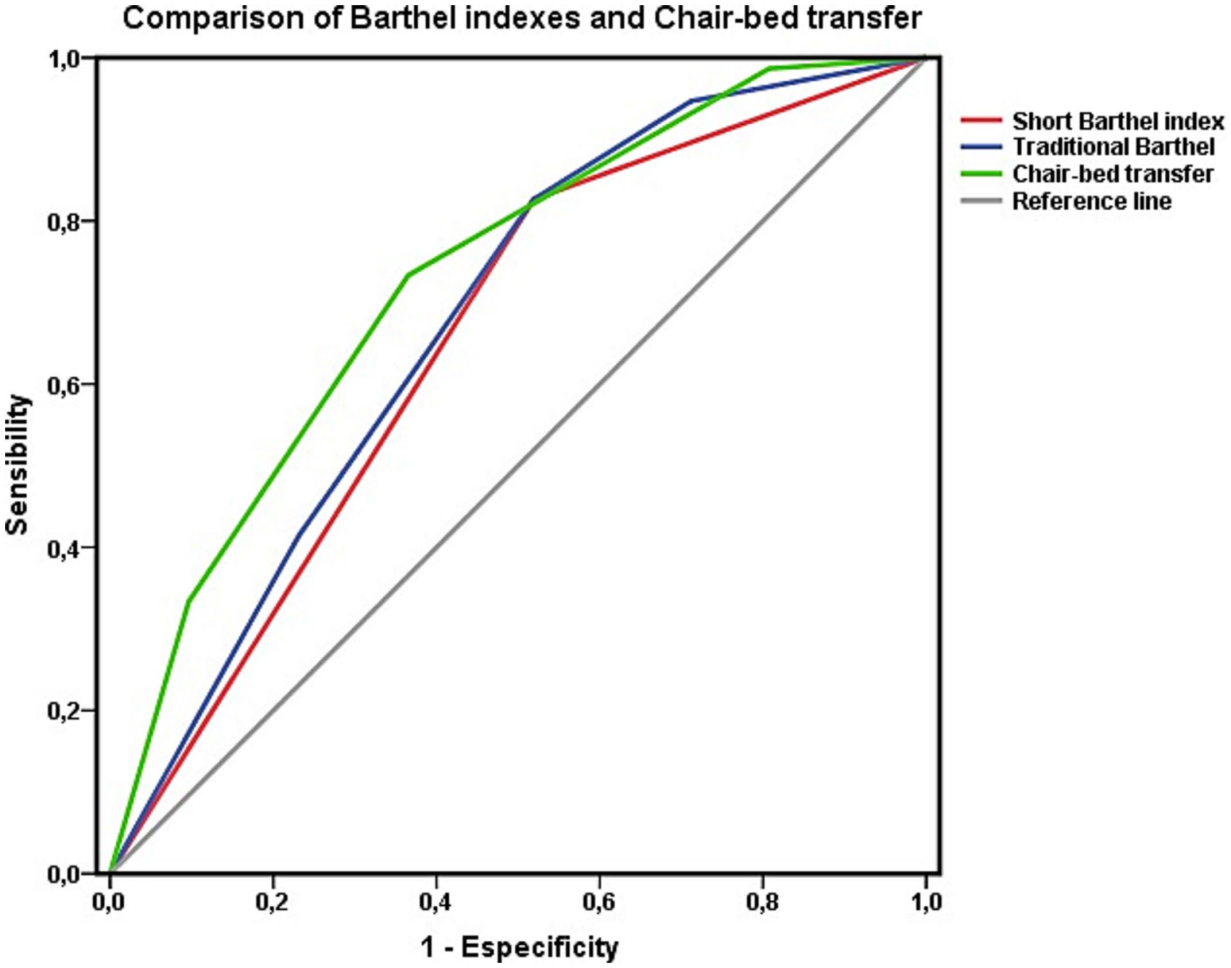
Areas under the ROC curve of the Barthel index estimated using the traditional four-level classification and the abbreviated two-level classification with respect to survival at 3 years. Comparison with the item “chair-to-bed transfer. Data for the whole cohort.

Finally, as shown in Figure 4, the areas under the curve of both the Barthel Index model and the “chair-to-bed transfer” factor were analyzed for the entire cohort, without excluding any patients, to determine their predictive capacities in common clinical practice. The four classification levels of both the chair-to-bed transfer factor and the Barthel Index are represented in this figure.

**Figure 5:**
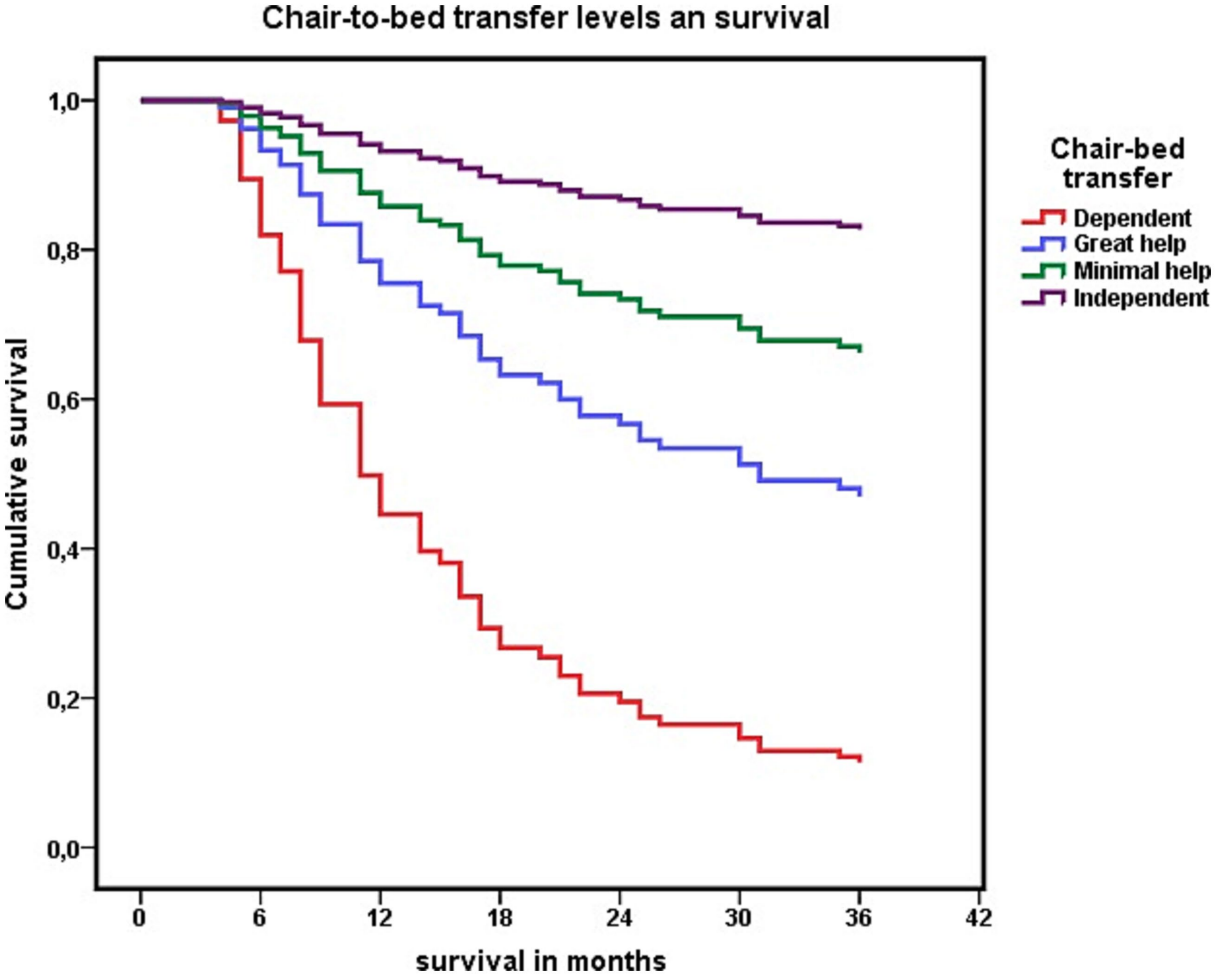
Cox regression analysis of the association between each level of dependency to perform “chair-to-bed transfers,” and survival at 3 years.

A survival analysis was performed via Cox regression to complete this analysis. This analysis revealed that being independent for “chair-to-bed transfers” was associated with a lower risk of mortality at the 3-year follow-up (HR 0.459; CI 0.331–0.638). The risk of mortality increased as dependence on this activity increased, and mortality associated with needing great assistance to perform “chair-to-bed transfers” was also relevant. No significant differences were observed between the “independent” and “need minimal assistance” levels (Figure 5).

These results led us to consider dichotomizing the “chair-to-bed transfer” activity into two profiles: 1: “being dependent or needing great assistance from others to perform chair-to-bed transfers” and 2: “being independent or requiring minimal assistance to perform chair-to-bed transfers.”

Dichotomizing the activity “chair-to-bed transfer” into the two options described, being “dependent or needing great assistance to perform chair-to-bed transfers,” was associated with an increased risk of mortality (HR 2.957; CI 1.678–5.211). Among the participants who died in these 3 years, 63.5% (*n* = 33) were “dependent or needed great assistance to perform chair-to-bed transfers.” Among those who were “independent” for this activity or “required minimal assistance” (*n* = 74), 36.5% (*n* = 19) died.

When this result was cross-checked with the second diagnostic test, the summary version of the Barthel index, among people with severe functional dependence (*n* = 28; Barthel Index score < 40 points) and “dependence or need for great assistance in chair-to-bed transfers,” 75% (*n* = 21) died in the 3 years (2020–2023). Among people with moderate functional dependence (*n* = 25; Barthel Index score of 40–60 points), 48% (*n* = 12) of those with “dependence or the need for great assistance” for chair-to-bed transfers died during this three-year period, whereas 23.4% (*n* = 15) of those who were not dependent or required minimal assistance died (OR 0.332; CI 0.125–0.879).

The distribution of mortality in relation to the level of functional dependence assigned according to the classic Barthel Index was also relevant. A total of 28.3% (*n* = 15) of the persons with “dependence or the need for great assistance for chair-to-bed transfers” had moderate functional dependence (Barthel Index score of 40–55 points). In addition, 18.9% (10) of the participants had a mild level of functional dependence (Barthel Index score of 60 points). Among those with moderate functional dependence who were also dependent or needed great assistance for “chair-to-bed transfers,” 53.3% (*n* = 8) died. This mortality was lower among participants who, despite being dependent on “chair-to-bed transfers,” had an overall level of dependence classified as mild, a group in which 40% of the participants died (*n* = 4).

It was also relevant to know whether this mortality was influenced by nutritional status, age, disease burden, mobility, or social support. With respect to nutritional status, no differences were observed in relation to BMI (Mann–Whitney U, z = −0.849, *p* = 0.396) between those who were “dependent or needed great help to perform the chair-to-bed transfer” (28.22 ± 5.25, range 15.77–41.42) and those who were “independent or required minimal help” (28.83 ± 4.30, range 15.06–42.67).

Age did not influence greater or lesser dependence to perform chair-to-bed transfers (Mann–Whitney U z = −1.690; *p* = 0.091). The mean age among those who presented “dependence or needing great help to perform chair-bed transfers” was 87.57 ± 6.7 years, with a range between 68 and 102 years, while among those who were “independent or needed minimal help,” this mean was 85.96 ± 5.9 years, with a range between 66 and 99 years.

Nor were differences associated with disease burden observed (OR 0.875; CI 0.396–1.935), with 28.3% (*n* = 15) of those with “dependence or needing great assistance to perform the chair-bed transfer” and 25.7% (*n* = 19) of those who were “independent or required minimal assistance” having five or more chronic diseases.

Regarding mobility, in June 2020, 69.8% (*n* = 37) of people who were “dependent or needed major assistance” to perform chair-to-bed transfer were living homebound, while 67.6% (*n* = 50) of people who were “independent for this activity or needed minimal assistance” maintained the ability to leave home (OR 0.208; CI 0.097–0.445). Greater independence in performing chair-to-bed transfers was associated with a lower probability of living homebound. Extending this analysis (Table 3), the Barthel index activity that presented the strongest relationship between a higher level of dependence and a higher probability of living homebound was chair-to-bed transfer.

**Table 3:** Mobility, estimated by the ability to leave home versus living homebound, and functional dependence to perform basic activities of daily living before the COVID-19 pandemic confinement.

Barthel index activities and mobility before covid-19 confinement					
Activity	Level	Leaves home	Homebound	Test	Statistic
Chair-bed transfers^1^	Unable	4 (4.9%)	7 (15.6%)	*χ*^2^ = 13.619	*p* = 0.003
	Major help	21 (25.6%)	21 (46.7%)		
	Minor help	32 (39%)	12 (26.7%)		
	Independent	25 (30.5%)	5 (11.1%)		
Dressing-undressing^1^	Dependent	21 (25.6%)	19 (42.2%)	*χ*^2^ = 4.212	*p* = 0.122
	Needs help	38 (46.3%)	14 (31.1%)		
	Independent	23 (28%)	12 (26.7%)		
Mobility on level surfaces^1^	Immobile or < 50 yards	13 (15.9%)	13 (28.9%)	*χ*^2^ = 4.793	*p* = 0.091
	Wheelchair independent or walks with help of one person	55 (67.1%)	29 (64.4%)		
	Independent	14 (17.1%)	3 (6.7%)		
Up-down stairs^1^	Unable	43 (52.4%)	36 (80%)	*χ*^2^ = 9.388	*p* = 0.009
	Needs help	26 (31.7%)	6 (13.3%)		
	Independent	13 (15.9%)	3 (6.7%)		
Feeding^2^	Unable	9 (11%)	8 (17.8%)	*χ*^2^ = 1.161	*p* = 0.560
	Needs help	14 (17.1%)	7 (15.6%)		
	Independent	59 (72%)	30 (66.7%)		
Bathing^2^	Dependent	58 (70.7%)	34 (75.6%)	*χ*^2^ = 0.339	*p* = 0.561
	Independent	24 (29.3%)	11 (24.4%)		
Grooming^2^	Needs help	35 (42.7%)	26 (57.8%)	*χ*^2^ = 2.652	*p* = 0.103
	Independent	47 (57.3%)	19 (42.2%)		
Bowels^2^	Incontinent	14 (17.1%)	12 (26.7%)	*χ*^2^ = 3.874	*p* = 0.144
	Occasional	15 (18.3%)	12 (26.7%)		
	Continent	53 (64.6%)	21 (46.7%)		
Bladder^2^	Incontinent	23 (28.1%)	21 (46.7%)	*χ*^2^ = 5.233	*p* = 0.073
	Occasional	43 (52.4%)	15 (33.3%)		
	Continent	16 (19.5%)	9 (20%)		
Toliet use^2^	Dependent	12 (14.6%)	11 (24.4%)	*χ*^2^ = 4.136	*p* = 0.126
	Needs some help	26 (31.7%)	18 (40%)		
	Independent	44 (53.7%)	16 (35.6%)		

1: tasks associated with mobility. 2: tasks associated with self-care. Both, according to Granger’s classification.

Finally, 60.4% (*n* = 32) of those with “dependence or needing great help to perform the chair-bed transfer” lived with their children, 20.8% (*n* = 11) lived with people other than their children, and 21.3% (*n* = 10) lived independently with their partner. These percentages were 33.8% (*n* = 25), 16.2% (*n* = 12), and 78.7% (*n* = 37), respectively, among those who were “independent or required minimal assistance” (*χ*^2^ = 13.305; *p* = 0.001).

The Barthel Index model with a cut-off score of 40 points and two categories, severe (Barthel Index score < 40 points) and moderate (Barthel Index score of 40–60 points) dependence, was used as a reference for this comparison. According to the results of this Barthel Index model, from 2020 to 2023, 48.1% (*n* = 25) of all persons with functional dependence who died had severe functional dependence, and 51.9% (*n* = 27) had moderate functional dependence. Within the group of persons with moderate functional dependence who died, 44.4% (*n* = 12) were dependent or needed great assistance for “chair-to-bed transfers.” Severe functional dependence (Barthel Index score < 40 points) was associated with an increased risk of mortality (HR 2.227, CI 1.514–3.276). This risk was lower than that observed for “being dependent or requiring great assistance for chair-to-bed transfers.”

With this new model being used as a diagnostic test to assess the risk of mortality (Table 4), lower results were obtained when “dependence for chair-to-bed transfers” and “need for great assistance for chair-to-bed transfers” were included in the same variable. The performance was highest when only the “dependence for chair-to-bed transfers” was included in the model.

**Table 4:** Analysis of the “chair-to-bed transfer” indicator as a diagnostic test for mortality risk.

Evaluation of the Barthel index and chair-to-bed transfer indicators^1^				
Chair-to-bed transfer and survival in 2023				
Chair-to-bed transfer	Status as of June 2023	Total dependence or the need for great assistance	Independence for chair-to-bed transfer^3^	Statistical test
	alive	20	25	*χ*^2^ = 16.050 *p* < 0.001
	deceased	33	5	

Evaluation of the chair-to-bed transfer indicator as a test of three-year mortality risk			
Test	Value	95% confidence interval	
		Lower limit	Upper limit
Sensibility	0.62	0.49	0.75
Specificity	0.83	0.70	0.97
Predictive value (+)	0.87	0.76	0.98
Predictive value (−)	0.56	0.41	0.70
Likelihood ratio (+)	3.74	1.63	8.54
Likelihood ratio (−)	0.45	0.31	0.66
Posttest probability (+)	0.79	0.62	0.90
Posttest probability (−)	0.31	0.24	0.40

The Barthel Index and survival in 2023				
Barthel index	Status as of June 2023	Severe Dependency(Barthel Index score < 40 points)	Moderate Dependency (Barthel Index score of 40–60 points)^3^	Statistical test
	Alive	13	49	*χ*^2^ = 17.942 *p* < 0.001
	Deceased	25	15	

Evaluation of the Barthel index as a three-year mortality risk test			
Test	Value	95% confidence interval	
		Lower limit	Upper limit
Sensibility	0.66	0.51	0.81
Specificity	0.77	0.66	0.87
Predictive value (+)	0.63	0.47	0.78
Predictive value (−)	0.79	0.69	0.89
Likelihood ratio (+)	2.81	1.70	4.62
Likelihood ratio (−)	0.45	0.28	0.72
Posttest probability (+)	0.74	0.63	0.82
Posttest probability (−)	0.31	0.22	0.42

Comparative analysis with respect to the Barthel Index (gold- standard). 1: Analysis was performed using the following criteria: “Exposed”: independence for “chair-to-bed transfer” or a Barthel Index score of 60 points. “Not exposed”: dependence or the need for great assistance for “chair-to-bed transfers” or total dependence (Barthel Index score < 20 points). Positive test: death. Negative test: survival. 2: includes people who were independent in performing the “chair-to-bed transfer” or who required minimal assistance. 3: Moderate dependency: only included people with moderate dependency who were not dependent and did not require great assistance for chair-to-bed transfers.

However, these results varied only slightly from those obtained with the gold standard, the summarized version of the Barthel Index, which has two levels of dependence (severe–moderate) and maintained statistical significance. To perform these analyses, in the group of patients with mild functional dependence (Barthel Index score of 60 points), 24 participants were excluded for “needing great assistance for ‘chair-to-bed transfer.’ One person with dependence for “chair-to-bed transfers,” who had a moderate level of functional dependence, was also excluded. Thus, in this group, which corresponded to the “unexposed” group, there were no “exposed” participants included.

When assessing their predictive ability, the area under the ROC curve for the dichotomized “chair-to-bed transfer” variable was 0.684 (CI 0.588–0.780; *p* < 0.001). For the dichotomized version of the Barthel Index, and in the circumstances described, the area was 0.708 (CI 0.601–0.814; p < 0.001). When the areas under the ROC curve were compared via the DeLong method, the differences between the areas of the dichotomized “chair-to-bed transfer” and dichotomized Barthel index variables were not significant (*χ*^2^ = 0,004; *p* = 0,948).

Bivariate analysis revealed that the variables “sex” (HR 0.486; CI 0.272–0.869) and “level of economic income” (HR 2.376; CI 1.357–4.161) were significantly related to survival at the three-year follow-up. Thirty-five percent (*n* = 35) of the females and 63% (*n* = 17) of the males died during this period; females had a lower mortality risk. However, sex was not associated with greater or lesser dependence for “chair-to-bed transfers” (OR 0.869; CI 0.369–2.047) or with greater or lesser ADL dependence, as assessed by the Barthel Index (OR 1.018; CI 0.402–2.580).

Using multinomial regression, when both variables were included in the model, the constant was no longer significant. Introducing the “chair-to-bed transfer” activity and the “level of economic income” into the dichotomized version of the model, the Cox and Snell R2 value showed that the model explained 17.3% of the variance in survival, and Nagelkerke’s R2 showed that the model explained 23.4% of the variance. The concordance index C was 0.736. Finally, the Hosmer–Lemeshow test was used to assess the goodness-of-fit of the model, and the results were as follows: *χ*^2^ = 0.709 and *p* = 0.702. This model had a sensitivity of 73.3% and a specificity of 63.5%.

By substituting the “chair-to-bed transfer” activity for the Barthel Index and following the same evaluation process, the Cox and Snell R2 value for the Barthel Index showed that the model explained 21.1% of the variance in survival, while Nagelkerke’s R2 showed that the model explained 28.6% of the variance. The concordance C index was 0.761, and the Hosmer–Lemeshow test used to assess the goodness-of-fit of the model yielded the following results: *χ*^2^ = 0.794 and *p* = 0.672. This model had high sensitivity (91.9%) and low specificity (47.5%).

In addition, a comparative analysis was performed between the Barthel Index and the “chair-to-bed transfer” activity, assessing survival in six-month periods, which revealed that the predictive capacity of the “chair-to-bed transfer” item as a risk factor progressively increased, and at 36 months, it surpassed that of the Barthel Index (Table 5). Although the level of association of both indicators with survival increased over time, “chair-to-bed transfers” did so to a greater extent.

**Table 5:** Level of dependence, dependence for “chair-to-bed transfers,” and survival in the three-year follow-up period.

Chair-to-bed transfers, level of dependence, and survival at the 3-year follow-up			
Time	Risk factors	Odds ratio (Confidence Interval)	*p* value
12 months	Chair-to-bed transfers	0.486 (0.201–1.178)	0.106
	Barthel Index	0.300 (0.121.0.741)	0.007
18 months	Chair-to-bed transfers	0.389 (0.179–0.846)	0.016
	Barthel Index	0.271 (0.120–0.612)	0.001
24 months	Chair-to-bed transfers	0.334 (0.157–0.711)	0.004
	Barthel Index	0.239 (0.107–0.534)	<0.001
30 months	Chair-to-bed transfers	0.265 (0.125–0.562)	<0.001
	Barthel Index	0.228 (0.102–0.510)	<0.001
36 months	Chair-to-bed transfers	0.209 (0,098–0.449)	<0.001
	Barthel Index	0.226 (0.101–0.508)	<0.001

Comparative analysis.

On the other hand, within the analysis using ROC curves, when differentiating between the group of activities associated with self-care and those related to mobility, dependence on activities involving mobility tended to be associated with lower survival.

**Figure 6:**
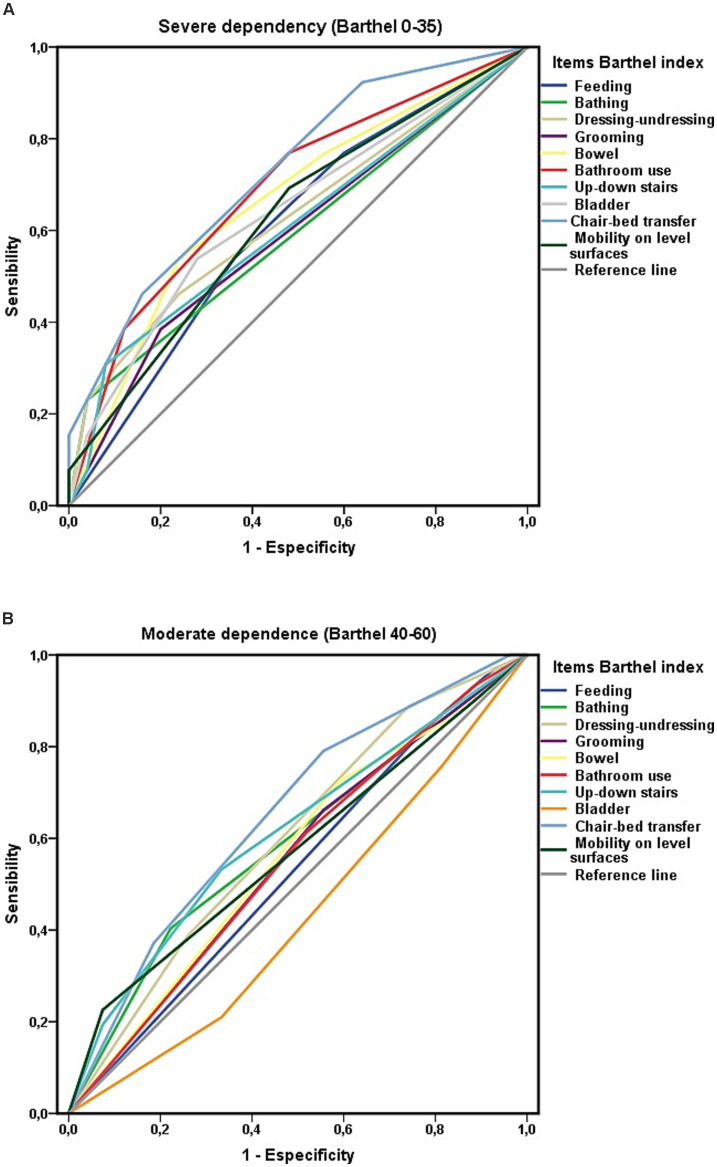
**(A)** Areas under the curve ROC of the Barthel index items and survival at 3-year follow-up among people with severe ADL dependence (Barthel less than 40). **(B)** Areas under the curve ROC of the Barthel index items and survival at 3-year follow-up among people with moderate ADL dependence (Barthel 40–60).

The study’s objectives also included assessing whether the level of dependence influenced the results. For this reason, in parallel, the area under the curve for each of the activities included in the Barthel index was analyzed via ROC curves separately for each level of dependency: moderate (Barthel Index score of 40–60 points) and severe (Barthel Index score < 40 points). For both severely dependent persons (Figure 6A) and moderately dependent persons (Figure 6B), the only item that showed statistical significance in relation to survival was “chair-to-bed transfers.”

Extending the analysis by the level of ADL-dependency, greater independence in performing chair-to-bed transfers was associated with a lower risk of mortality, both in individuals with severe (HR 0.492; CI 0.290–0.865) and moderate (HR 0.574; CI 0.355–0.927) ADL-dependence. Among the participants with moderate ADL dependence, 48% (*n* = 12) of those who were “dependent or required great assistance for the “chair-to-bed transfers” (*n* = 25) died, whereas 23.4% (*n* = 15) of those who could perform this activity independently or with minimal help (*n* = 64) died. Among the group of persons who were “dependent or required great assistance to perform chair-to-bed transfers” who died (n = 33), 36.4% (*n* = 12) had moderate ADL dependence, and 63.6% (*n* = 21) had severe ADL dependence.

To complete the objectives of the study, the influence of economic level on the results was analyzed. In this sense, a lower level of economic income was associated with a higher level of dependence (OR 2.524; CI 1.153–5.524) and with higher mortality (HR 2.376; CI 1.357–4.161). On the other hand, among those who had “dependence or need for great assistance” to perform the chair-bed transfer, 52.8% (*n* = 28) had severe functional dependence, and 47.2% (*n* = 25) had moderate dependence. Finally, greater dependency to perform chair-bed transfers was associated with an increased mortality risk (HR 2.957; CI 1.678–5.211). When using Cox regression and a proportional hazards model, both variables, economic level and level of dependence for chair-bed transfers, were included. Being dependent on chair-bed transfers and having a lower economic level implied a higher mortality risk at 3 years (HR 2.999; CI 1.699–5.2921).

Extending this analysis for each economic level group, this association was maintained. When the economic income was less than 11,200 euros/year, most of the people who were dependent on chair-bed transfers (*n* = 27) died (77.8%; *n* = 21), while when they were “independent or required minimal assistance” (*n* = 33), only 33.3% (*n* = 11) died (OR 0.143; CI 0.045–0.456). This result was also present when the income was higher than 11,200 euros/year, with 46.2% (*n* = 12) of those who were “dependent or required great assistance for chair-bed transfer” (*n* = 26) dying within this economic level, and 19.5% (*n* = 8) of those who were “independent or required minimal assistance” (*n* = 41; OR 0.283; CI 0.095–0.842).

**Figure 7:**
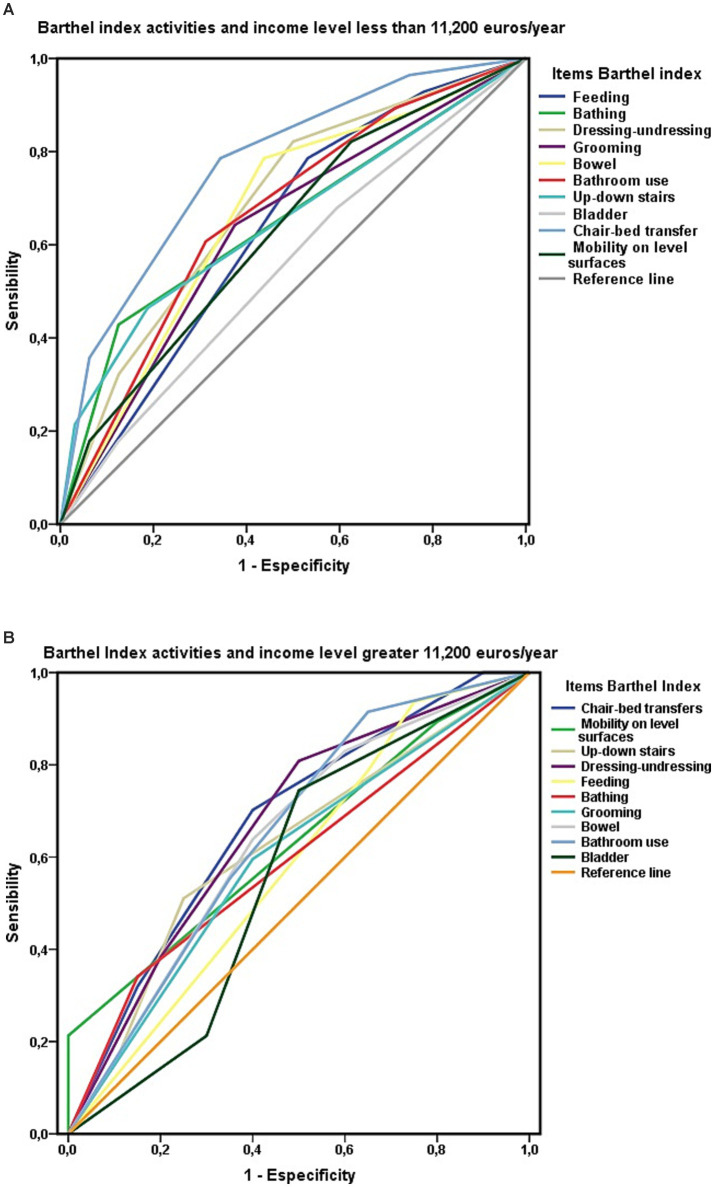
**(A)** ROC curve for the variables level of dependence and Barthel index item “chair-to-bed transfer” in people with ADL dependence and economic income less than 11,200 euros/year. **(B)** ROC curve for the variables’ level of dependence and Barthel index item “chair-to-bed transfer” in people with ADL dependence and economic income greater than 11,200 euros/year.

The AUC of each item of the Barthel Index was analyzed via ROC curves according to the level of economic income. In the group of people with functional dependence who had an income of less than 11,200 euros/year, the “chair-to-bed transfer” item reached a “good” level of discrimination in relation to survival at 3 years of follow-up (Figure 7A). When there was a higher economic level, the association of the Barthel Index items with survival visualized through the ROC curves was weaker (Figure 7B).

Finally, in the *post hoc* analyses, the “chair-to-bed transfers” item explained 17.7% of the variance in survival. Differences among the four levels of dependency of the classic Barthel Index model explained 11.8% of the variance in survival, whereas differences between the two levels of the abbreviated Barthel Index model explained 7.5% of the variance. Dichotomizing the levels of the “chair-to-bed transfer” activity meant that the percentage of the variance that explained the differences in this variable decreased to 13.5% (Table 6). Age, being over 90 years old, and being widowed did not influence these results. Mortality was higher among men in this cohort.

**Table 6:** *Post hoc* regression analysis and survival at the three-year follow-up.

*Post-hoc* regression analysis and survival at the three-year follow-up							
Variable	R square	Adjusted R squared	Std. Error of the Estimate	t	Sig.	95% Confidence interval for B	
						Lower	Upper
Standard Barthel Index^1^	0.118	0.111	0.465	−4.096	<0.001	−0.242	−0.084
Summarized Barthel Index^2^	0.075	0.067	0.477	−3.178	0.002	−0.497	−0.116
Standard Chair-to-bed transfer^3^	0.177	0.170	0.450	−5.178	<0.001	−0.311	−0.139
Dichotomized chair-to-bed transfer^4^	0.135	0.128	0.461	−4.410	<0.001	−0.530	−0.202
Income level	0.057	0.049	0.481	−2.745	0.007	−0.404	−0,065
Sex	0.054	0.047	0.482	2.675	0.008	0.073	0.487
Educational level	0.036	0.028	0.487	2.151	0.033	0.023	0.554
Age	0.021	0.013	0.491	1.620	0.108	−0.02	0.025
Age over or under 90 years	0.027	0.019	0.489	1.853	0.066	−0.012	0.353
Marital status widowed	0.007	−0.001	0.494	−0.911	0.364	−0.265	0.098
Disease burden	0.006	−0.002	0.494	0.843	0.401	−0.113	0.280
Polymedicate	0.020	0.012	0.491	1.580	0.117	−0.062	0.552
Mobility BC^5^	0.035	0.027	0.487	2.124	0.036	0.013	0.371
Mobility AC^6^	0.103	0.096	0.469	3.793	0.001	0.151	0.481
Housing situation^7^	0.055	0.046	0.482	2.447	0.016	0.044	0.420
Community living^8^	0.023	0.015	0.490	1.722	0.087	−0.016	0.224

1: Classical Barthel Index with four levels of dependency: mild, moderate, severe, and total. 2: Barthel Index with two levels of dependency: moderate and severe. 3: Chair-to-bed transfer activity with four levels of dependency. 4: Simplified chair-to-bed transfer activity with two levels of dependency: a: dependent or requires great assistance for chair-to-bed transfers. b: Independent or requires minimal assistance for chair-to-bed transfers. 5: Mobility BC: leaves home versus homebound before COVID-19 confinement. 6: Mobility AC: leaves home versus homebound after COVID-19 confinement. 7: Housing situation: Live independently or live with children. 8: Lives independently, lives with children, or lives with others.

When sex and the level of dependency were included in the model, 24.7% of the variance in survival was explained. However, when sex, the level of dependency, and the level of income were included in the model one by one, the variable level of dependency no longer showed statistical significance. Significance was observed when sex and income level were included in the model, which explained 25% of the variance in survival. These results are summarized in Table 7.

**Table 7:** Survival at the three-year follow-up.

*Post-hoc* regression analysis and survival at the three-year follow-up								
Model	R square	Adjusted R square	Std. Error of the Estimate	Model	*t*	Sig.	95% Confidence interval for B	
							Lower	Upper
Model 1 Summary								
1	0.054	0.047	0.482	1^a^ (Constant):	8.000	0.000	0.806	1.335
				Sex	2.675	0.008	0.073	0.487
2	0.175	0.162	0.452	2^b^ (Constant):	9.236	0.000	1.209	1.868
				Sex	2.930	0.004	0.093	0.481
				Barthel level	−4.271	0.000	−0.242	−0.089
3	0.247	0.229	0.434	3^c^ (Constant):	10.117	0.000	1.467	2.181
				Sex	2.695	0.008	0.068	0.442
				Barthel level	−2.231	0.027	−0.179	−0.011
				Chair-to-bed transfer	−3.422	0.001	−0.260	−0.069
Model 2 Summary								
1	0.054	0.047	0.482	1^d^ (Constant)	8.000	0.000	0.806	1.335
				Sex	2.675	0.008	0.073	0.487
2	0.110	0.096	0.469	2^e^ (Constant):	7.804	0.000	1.067	1.791
				Sex	2.720	0.007	0.075	0.479
				Income level	−2.788	0.006	−0.398	−0.068
3	0.250	0.232	0.433	3^f^ (Constant):	9.716	0.000	1.553	2.347
				Sex	2.565	0.012	0.055	0.428
				Income level	−2.345	0.021	−0.336	−0.028
				Chair-to-bed transfer	−4.796	0.000	−0.287	−0.119

Post hoc tests were performed via linear regression analysis in relation to survival in June 2023. Stepwise regression analysis. Predictors in Model 1: a: Predictors: Sex. b: Predictors: sex and the Barthel Index score. c: Predictors: sex, Barthel Index score, and chair-to-bed transfers. Predictors in Model 2: d: Predictors: Sex. e: Predictors: sex and income level. f: Predictors: Sex, income level, and chair-to-bed transfer. Dependent variable: survival in June 2023.

## Discussion

This study was conducted in a population cohort with functional dependence on basic ADLs that lived either independently or with a partner, adult children, or other people in the community.

In this cohort, dependence on chair-to-bed transfers was an independent risk factor for mortality. Dependence on chair-to-bed transfers involved a lifestyle that has come to be called “armchair-bed living.” The results of this study suggest that this mode of living is, in fact, a morbid state, which at the clinical level could be equated to being “pre-bedridden.” “Armchair-bed living” was characterized by a relevant functional limitation that required assistance from others for minimal transfers within the home, a situation that conditioned a higher probability of living homebound and a higher probability of having to live with children or others, and that was associated with high mortality. This probability, however, was not associated with age, disease burden, or nutritional status in this study. Doing “armchair-bed living,” that is, being pre-bedridden, would, in addition, aggravate the mortality risk associated with dependence on other Barthel index activities and would increase the mortality risk among persons with moderate functional dependence.

On the other hand, the loss of the ability to get out of bed autonomously is what ultimately determines the loss of functional independence and, regardless of the ability to perform other activities, forces them to be cared for by others. It is, therefore, the activity of the Barthel index that probably reflects, to a greater extent, the dependence of dependent persons (33).

Detecting vulnerable populations is a priority objective in health planning. This study suggests that a way of life, the “armchair-bed life,” is a morbid state with high mortality that should be acted upon. The first step is to recognize this situation and establish the necessary measures for its detection. For this purpose, a test is available based on the “chair-bed transfer” activity of the Barthel index, which has been shown in this study to work well as a screening test to assess the risk of mortality, its results being superior to the method used as the gold standard, the Barthel index.

Furthermore, these results show that the Barthel index may have blind spots, depending on its design, and that the index may be used for purposes it was not designed for. The results presented here suggest that the Barthel index should be modified, adapting it to the new objectives of health planning and incorporating the assessment of mortality risk into the assessment of functional capacity.

Historically, the Barthel Index was designed to assess capabilities, not risk or survival. However, these capacities are associated with survival. This study delved into the nature of these associations.

Consistent with what has already been reported in other studies, functional dependence in performing basic ADLs was associated with high mortality in this cohort, and this mortality increased with an increasing level of dependence (22, 23). The two modalities of classification of the Barthel index, the traditional four-level model and the simplified two-level model, showed a similar capacity for determining mortality risk; thus, in relation to health planning, both models are comparable.

Essentially, all the activities assessed by the Barthel Index were associated with survival, showing the usefulness of this index as a predictor of mortality risk. However, nuances in this association were observed. Activities associated with mobility showed a greater association with survival than activities associated with self-care, justifying the classification proposed by Granger (7).

One activity stood out in particular: “chair-to-bed transfers.” Nine out of 10 people who were dependent on chair-to-bed transfers died in the 3 years. No other activity in the Barthel Index was associated with this level of mortality. Dependence on “chair-to-bed transfers” implied a high risk of mortality. This translated into two out of every three persons who died in this cohort being dependent or requiring great assistance for “chair-to-bed transfers.”

It also modulated survival when, in addition, one was dependent on any of the other activities included in the Barthel index, increasing mortality for that other activity. However, mortality was much lower for these same activities when the patient was not dependent on chair-to-bed transfer. Dependence on chair-to-bed transfer was an additional risk factor.

On the other hand, the risk of mortality associated with dependence on performing the “chair-to-bed transfer” increased as the follow-up period progressed until it surpassed that reflected by the Barthel index at 3 years. At the same time, the risk also increased as dependence to perform the “chair-to-bed transfer” increased. It was observed that this risk was particularly high when great assistance was required to perform the “chair-to-bed transfer” so that if both items were combined, it was observed that two out of every three persons who died in this three-year period were persons who were “dependent or required great assistance to perform the chair-to-bed transfer.” Being dependent or needing great assistance for chair-to-bed transfers was a risk factor for mortality in individuals with both severe and moderate ADL dependence.

Within the group of people with moderate dependence in this study, the presence of “dependence or the need for great assistance for chair-to-bed transfers” increased the potential risk of mortality estimated after performing an assessment of functional capacity via the Barthel Index and obtaining a classification of “moderate functional dependence.” Showing one of the possible blind spots of the Barthel index.

These results suggested that the “chair-to-bed transfer” variable could be used as a dichotomous (dependent-independent) screening test to assess mortality risk, as it effectively discriminates functional capacity. To validate its effectiveness, we compared it with the Barthel index, which is the gold standard for assessing functional capacity. For this comparison, we established two categories for each diagnostic test: being dependent in the “chair-to-bed transfer” activity or having total dependence (Barthel <20), and being independent in the “chair-to-bed transfer” activity or having a Barthel index score of 60. Since the study involved a population diagnosed with functional dependence, a Barthel score of 60—indicative of mild functional dependence according to National Health System protocols—was considered the nearest threshold to independence.

The result of this contrast was that the chair-to-bed transfer factor showed a good degree of accuracy in the discrimination of mortality risk and predictive ability, compared to the Barthel index, which only achieved a regular grade in both aspects.

This nuance may have contributed to the fact that the concordance C index showed a greater probability of predicting death for a person with chair-to-bed transfer dependency than for a person with a total functional dependency, according to the Barthel index. The C-index was closer to the chair-to-bed transfer factor to the established value for a diagnostic test, a value of 0.85.

However, when the Hosmer–Lemeshow test was used, the calibration of the Barthel Index as a diagnostic test for assessing mortality risk was superior to that of the chair-to-bed transfer factor.

Finally, and because of the above, Nagelkerke’s R2 value showed that the chair-to-bed transfer factor explained more than half of the variance in survival. However, the Barthel Index explained only slightly more than a third of this variance.

In summary, the “chair-to-bed transfer” factor showed functioning as a screening test to assess the risk of mortality that can be considered good, with results superior to the method used as a gold standard, the Barthel index.

Analyzing the differences between the two tests, the Barthel index includes 10 activities, and each activity has a dependency gradient, from “independent” to “dependent” for that activity. Each position on that gradient is assigned a value. When the sum of the values obtained for a particular person is 60 or lower, the person has a functional dependency to perform basic activities of daily living. By representing an average value of functional dependence, the Barthel index “hides” the situation of being dependent or independent for some activities, and the person may be dependent on performing the chair-bed transfer but independent for eating or toileting. This is another blind spot of the Barthel index, making it possible that clinically relevant dependencies may remain invisible because they do not reach 60 points through the rest of the activities. This situation adds value to the use of the “chair-bed transfer” activity as a mortality screening test since it is not subject to an average value. It probably justifies that, among people with moderate functional dependence, those with chair-bed transfer dependence had a higher mortality.

Compared to the Barthel index, the “chair-to-bed transfer” test offers the advantages of the simplicity of the method, its immediate availability without the need for a questionnaire, the short time required to perform it, and the fact that it can be easily evaluated by minimally trained personnel. Its routine setting of use would be the evaluation of the risk of mortality in people with functional ADL dependence. However, it can also be useful in multiple other clinical situations that involve a limitation to performing chair-to-bed transfer, from assessments at hospital admission or discharge, major depression, cognitive impairment, or other clinical situations.

All these reasons justify our recommendation to include this test in routine clinical practice in parallel to the Barthel index.

As a limitation of this comparison between the diagnostic tests, the Barthel Index, dichotomized into total functional dependence and mild functional dependence, included patients with chair-to-bed transfer dependence among the group of people with total functional dependence. Analyzing this situation by means of ROC curves, it was observed that being dependent on performing chair-to-bed transfer contributed a relevant part of the area under the ROC curve of the Barthel index.

Therefore, this influence on the results was analyzed. There was a great difference in mortality between patients with “dependence or needing great assistance for chair-to-bed transfers” and those with “independence or needing minimal assistance” to perform this activity. In addition, there were likely differences in the evolutionary courses that people with functional dependence for basic ADLs ended up following, with differences marked by the situation of being dependent or not being able to perform “chair-to-bed transfers.”

In this context, and to solve this problem, a contrast was performed that only included patients who were not duplicated in each test. Using this criterion, the mortality risk remained higher than that provided by the Barthel index. This result made it possible to establish the validity of the dichotomous version of this test as a screening for mortality associated with dependence on the activity “chair-to-bed transfer.”

On the other hand, several factors that could have influenced these results were analyzed. Among the confounding factors that could affect the results, the economic level was analyzed. Simultaneously, and according to the data obtained in this study, mortality associated with dependence for “chair-to-bed transfers” was modulated by the level of economic income, with higher dependence being associated with a lower economic income level. However, for each economic income level, “dependence or needing great assistance for chair-to-bed transfers” also implied lower survival. In other words, the “chair-to-bed transfer” factor was present at any economic level, although its effect was greater with lower economic income levels.

However, the relevant role of the “chair-to-bed transfer” activity in relation to mortality probably encompasses more aspects than those analyzed in this study. Dependence for chair-to-bed transfers, together with the “dressing-undressing” activity, which, in this study, ranked second regarding the association with survival, has been independently associated with survival in studies of institutionalized persons with depressive symptoms (34). Depression is associated with increased mortality (35). Being dependent on the chair-to-bed transfer activity generally implied living homebound and living with children or other people, situations that could affect the emotional state of these people.

The assessment of the ability to perform “chair-to-bed transfers” has occasionally been used in clinical practice beyond its role within the Barthel index. In addition, this assessment has been used in multiple ways, such as detecting frailty in liver transplant patients (36) and in the evaluation and follow-up of patients who have suffered a stroke (37, 38). The assessment of dependence for “chair-to-bed transfers” has also been used as a criterion to establish which patients are likely to have a prolonged hospital stay (39).

In this study, the results were obtained in a population living in the community, which was followed for 3 years. The survival of individuals in this population was not affected by comorbidities or the number of drugs they were taking. In addition, the baseline values of the Barthel index are prior to confinement for the COVID-19 pandemic, which minimizes its impact on the results, and the people who did armchair-bed living after confinement were the same as those who did it before confinement.

For these reasons, it seems appropriate to review the role of the “chair-to-bed transfer” factor in the global assessment of functional capacity via the Barthel index, an assessment that should consider the risk of mortality inherent in being functionally limited in performing chair-to-bed transfers.

The Barthel Index was designed by Mahoney and Barthel in 1995 to evaluate the functional capacity of patients with neuromuscular and musculoskeletal problems (6). And to observe the evolution of this capacity over time. This tool was not initially designed to assess mortality risk, but it has been shown to have a clear association with mortality risk since its development. The indicators included in this index in the form of basic ADLs were included since consensus among its authors, so it is logical to observe differences in their associations with survival.

In 1995, the life expectancy of the population was lower than it is today, and the phenomenon of “population aging” did not yet exist. In both Latin societies (40) and many other societies (41), adult children play a key role in caring for their aging parents. However, this social model may be in crisis. A crisis in which the declining birth rate, together with working conditions that are not conducive to family reconciliation for older people, presents a future problem that needs to be addressed.

On the other hand, the increase in the number of people over 65 years of age has created a new group, the so-called “older adults” group, with influence at all levels. This situation has led to the concepts of healthy aging and quality of life being discussed in many organizations, ranging from the World Health Organization (42, 43) to the various health and social institutions of individual countries (44, 45).

With the evolution of society, new needs are developing (46). On the one hand, a solution to the problem of the decrease in the number of potential adult children caring for their older adult parents is needed. However, at the same time, the problem of achieving healthy aging in the face of a greater number of people with functional dependence must be solved due to the increasing number of older adult people. These needs represent challenges that will have to be addressed in the coming decades (31, 47, 48).

These challenges require answers. The foreseeable results can be anticipated by modifying the starting situations or the paths to be followed (49, 50). To this end, it is necessary to establish the patterns that make up the networks of each process (50, 51).

In this sense, being bedridden, the final situation of many dependent people is a factor that has already been shown to be associated with an increased risk of mortality (52).

Although the “being bedridden” variable was not analyzed in this study, this situation may explain why, among the totally dependent persons, most of those who died were not dependent on “chair-to-bed transfers.” They were probably bedridden patients. However, there is a process that occurs before a person becomes bedridden (53).

In this process, dependence on performing the “chair-to-bed transfer” would be the previous step before being bedridden. Making an armchair-bed life implies a greater degree of functional impairment and less mobility, which was made visible by a greater probability of living homebound or no longer leaving the house. In short, it was a situation equivalent to being pre-bedridden. This study contributes to the knowledge of the network associated with dependence. Of the steps that end up leading a person to be homebound and, finally, to end up bedridden. And to make visible a new group of patients, the pre-bedridden, who, together with the homebound, probably have a limited circle of social relations (54).

This previous step, making an armchair-bed life, was not associated in this study with age, nor with a greater burden of disease or different nutritional status, a result that suggests that other factors are the main factors associated with this situation. However, it was observed that most of these people ended up living with their children or with other people, showing the relevance of social support in these final stages of life. This cohabitation implied a greater probability of living homebound, a situation that has been associated with higher mortality (22, 23, 25–27).

However, more answers are needed. In addition to assessing functional capacity, the Barthel Index must adapt to the time. In relation to functional capacity, mortality risk must be measured not only globally but also by establishing which groups are more vulnerable, which activities are associated with greater risk, or, simply, which activities in the index may be independent risk factors related to mortality.

In this process, it will probably be necessary to include other activities. These include instrumental ADLs, which allow efficient stratification of risk beyond the functional capacity to perform basic ADLs. A holistic view is also likely necessary, including the networks present in different environments that activate/deactivate heuristics (55–57).

As a limitation of this study, in the post-hoc analyses performed, including the variables that showed statistical significance with survival, the models with the highest R^2^ were those shown. When other variables of clinical interest were included, such as disease burden or being polymedicated, it was not possible to increase this R^2^. Nor was it achieved by including social support (living independently versus living with children or versus living with other people) or the mobility indicator that represented having the ability to leave home versus living homebound. Therefore, most of the variance in mortality remains unexplained and is probably due to factors not captured in this study.

In addition, the population size probably did not allow us to obtain greater significance. Therefore, we propose that further studies be conducted to confirm the results obtained. On the other hand, the study was initiated under exceptional circumstances imposed by the COVID-19 pandemic. However, other circumstances support the idea that the COVID-19 pandemic did not have a relevant impact on these results. Among these circumstances are the duration of the study and the fact that the level of risk increased as the follow-up period increased, which removed the influence of the pandemic. But also, the response of this population to the pandemic, reflected in previous studies, implied an improvement in their functional capacity. Finally, the fact that much of the data predated the COVID-19 pandemic probably also minimized this effect.

### Proposal for a new classification of the Barthel index

The assessment of the mortality risk associated with functional dependence in this study showed that “dependence or the need for great assistance” for “chair-to-bed transfers,” assessed using the Barthel index, is associated with a higher mortality risk. This risk is present in any person with functional dependence and at all economic levels.

The results obtained show the existence of a possible blind spot in the Barthel index. The current classification does not allow for the detection of an increased risk of mortality among people who are “dependent or require great assistance for chair-to-bed transfers.” And it does not do so among people with moderate dependence on ADLs. In the Orcasitas cohort, one in five of the people who died in 2020–2023 had moderate functional dependence (Barthel Index score of 40–60 points) and “dependence or the need for great assistance for chair-to-bed transfers.” However, if we look at the deaths within the group of people with moderate functional dependence, almost half of those who died (44.4%) had “dependence or the need for great assistance for chair-to-bed transfers.” Finally, half of the people with “dependence or the need for great assistance for chair-to-bed transfers” who also had moderate functional dependence died in the 3 years (2020–2023). When the results are presented as a whole, the mortality in the group with moderate functional dependence is masked. Consequently, the level of risk and the corresponding level of intervention recommended for people with moderate dependence during health planning would not be adjusted to their true risk profile for those who are “dependent or require great assistance for chair-to-bed transfers.”

We consider that the evidence obtained through this study justifies a new classification for the Barthel index, as shown in Table 8. This classification expresses an assessment of functional capacity adjusted for mortality associated with this capacity. In this new classification, people with functional dependence who present “dependence or the need for great assistance for chair-to-bed transfers” are considered to have severe functional dependence, regardless of the functional capacity score obtained via the Barthel index.

**Table 8:** The Barthel index according to mortality risk.

New Barthel index
Barthel Index according to functional capacity and mortality risk
Severe dependence
Barthel index score of 0–35 points. Barthel index score of 40–60 points, with: “Dependence on other persons to perform chair-to-bed transfers” or“Need for great assistance in performing chair-to-bed transfers.”
Moderate dependence
Barthel index score of 40–60 points, with: “Independence for chair-to-bed transfers” or “Need for minimal assistance in performing chair-to-bed transfers.”

Proposed new classification of the Barthel index according to the mortality risk associated with functional dependence for the performance of basic activities of daily living.

This approach, equivalent to that used in the assessment of cardiovascular risk in people with diabetes mellitus, where having diabetes mellitus implies having a high cardiovascular risk, would also have implications at the level of social services in relation to the allocation of resources to help dependent persons.

Adopting this new classification, on the other hand, would also have clear utility in health planning, making it possible to allocate resources to people who need a higher degree of care, maintaining equity. If dependency on chair-to-bed transfers is an indicator that a patient’s hospital stay will be prolonged, once the patient is discharged, the time to reach his or her baseline state will also likely be prolonged. This aspect requires specific studies to confirm this, as well as the allocation of the necessary resources to facilitate recovery (39).

In primary care, the management of people with dependency falls mainly on nursing staff (58). A better knowledge of the functional dependency process and its risk factors (59–61) would make it possible for these professionals, through consensus with dependent persons and their relatives, to provide health education and take preventive actions against the development of dependency for chair-to-bed transfers (62).

At the same time, this new classification would also make it possible to prioritize the possible actions of physiotherapists and occupational therapists and direct them toward the population groups at greatest risk (50, 63, 64). This would favor the generation of greater evidence on the effectiveness of these actions in people who live at home with limitations in carrying out basic ADLs (65, 66). Although some actions have been shown to be effective in reducing functional limitations in people with functional dependence who are institutionalized (living in nursing homes) (67), there is still controversy in this regard (68).

Finally, as a limitation of the suggested approach, including “dependence for chair-to-bed transfers” and “requiring great assistance for chair-to-bed transfers” in the same variable reduced the test’s capacity to determine the risk of mortality, compared with assessing only “dependence for chair-to-bed transfers.” It would seem logical to have proposed including only the item “being dependent for chair-to-bed transfers” in the new Barthel Index classification and that presenting this dependence would be considered severe functional dependence, regardless of the Barthel score obtained.

However, our proposal has been argued throughout the paper. The “dependence-need for great assistance” is a risk factor for mortality, and most of the deaths in this study occurred in people who were “dependent or needed great assistance for chair-to-bed transfers.” Furthermore, although this decision results in a loss of discriminatory capacity, the discrimination capacity retained is still superior to that of the Barthel Index itself as a test for determining mortality risk.

## Conclusion

Aging is a natural process that occurs under very diverse conditions and depends on many factors. Recognizing these factors that generate differences makes it possible to reduce inequalities. This term, inequalities, acquires special relevance when the result is measured in terms of mortality.

Dependence for “chair-to-bed transfers” (in both continuous and dichotomous formats) has a significant negative relationship with survival, indicating that dependence on this activity is a risk factor for mortality. This dependence probably represents a morbid state, as does being permanently bedridden. This study lays the groundwork for defining that in people with functional dependence, the situation of leading an armchair-bed life is equivalent to being pre-bedridden and that this situation is a pathological state that increases the risk of mortality. Its recognition as such requires further studies to identify the characteristics of this state and its relationship with survival. The results obtained in this study suggest that persons with dependence or in need of great help to perform the chair-to-bed transfer activity should be followed up in a differentiated way, regardless of their score on the Barthel index.

A new test is proposed, based on the assessment of the ability to perform chair-to-bed transfers, which is simple to implement and which has shown its diagnostic effectiveness through both positive and negative likelihood ratios, as well as the rest of the parameters evaluated, which were favorable for its use as a discriminatory test for mortality risk compared to the Barthel index.

Implementing this dichotomous test is simple and does not require trained personnel. The results obtained suggest that it should be applied to older people with functional ADL dependence. However, the potential clinical contexts where it can be applied are multiple, including risk assessment at hospital admission, risk assessment of complications at hospital discharge, or assessment of the risk of mortality in older people with cognitive impairment or depression, requiring specific studies to support these recommendations.

The Barthel Index, in both formats, is also a significant predictor of mortality, with greater dependence associated with an increased risk of death.

However, the Barthel index has blind spots when, in addition to functional capacity, mortality risk is analyzed. Therefore, a new classification of the Barthel index is proposed, which includes both assessments, functional and mortality risk.

As a final recommendation, the presence of a new risk factor that generates differences in mortality among already vulnerable individuals justifies the recommendation of its use in the discrimination of mortality risk, both in national population-based studies or with large cohorts.

Similarly, we recommend that the new Barthel Index classification that we propose should be used in population-based studies, as well as in all studies targeting the population over 65 years of age. This classification would also be useful in all studies or situations in which functional dependence plays a role. The rationale for doing so is that it allows better discrimination of the mortality risk potentially associated with having functional limitations.

The recommendation to incorporate this new classification into protocols for the care of older persons is extended to health and social-health planning managers.

## Group members of the GIDO collaborative group (Orcasitas Dependency Research Group)

Vicente Martín Moreno, Orcasitas Health Care Center and i+12 Research Institute of the Doce de Octubre Hospital; GIDO group codirector, Madrid, Spain.

María Inmaculada Martínez Sanz, Orcasitas Health Care Center; GIDO group codirector, Madrid, Spain.

Amanda Martín Fernández, Polibea Concert, Madrid, Spain.

Sara Guerra Maroto, Orcasitas Health Care Center, Madrid, Spain.

Eva Sevillano Fuentes, Orcasitas Health Care Center, Madrid, Spain.

Elena Pérez Rico, Orcasitas Health Care Center, Madrid, Spain.

Irene Sánchez González, Orcasitas Health Care Center, Madrid, Spain.

Miriam Fernández Gallardo, Orcasitas Health Care Center, Madrid, Spain.

Julia Herranz Hernando, Orcasitas Health Care Center, Madrid, Spain.

María Palma Benítez Calderón, Orcasitas Health Care Center, Madrid, Spain.

Laura Calderón Jiménez, Orcasitas Health Care Center, Madrid, Spain.

Elena Sánchez Rodríguez, Orcasitas Health Care Center, Madrid, Spain.

Miguel Recuero Vázquez, Orcasitas Health Care Center and Nursing Home Care Unit of the Center Assistance Directorate, Madrid, Spain.

Helena Alonso Samperiz, Orcasitas Health Care Center, Madrid, Spain.

Irene León Saiz, Orcasitas Health Care Center, Madrid, Spain.

Juana Marcos Guerra, Orcasitas Health Care Center, Madrid, Spain.

## Data Availability

The original contributions presented in the study are included in the article/supplementary material, further inquiries can be directed to the corresponding author.
